# Polarizing Plates: Both Omnivores and Vegans Represent In-Group Foods With Eating Simulations

**DOI:** 10.1177/01461672231202276

**Published:** 2023-10-12

**Authors:** Tess Davis, Libby Harkins, Esther K Papies

**Affiliations:** 1 University of Glasgow, UK

**Keywords:** language, food, diet, grounded cognition, sustainability

## Abstract

In two pre-registered experiments, we assessed how people cognitively represent meat and plant-based foods, to examine processes underlying dietary polarization in society. Food descriptions from U.K.-based omnivores (*N_Exp. 1_* = 109; *N_Exp. 2_* = 436) and vegans (*N_Exp. 1_* = 111; *N_Exp. 2_* = 407) were coded for features about consumption and reward (e.g., “rich,” “indulgent,” and “treat”) or features independent of the consumption situation (e.g., “healthy,” “protein,” and “eco-friendly”). Participants used more consumption and reward features for diet-congruent dishes (meat dishes for omnivores and plant-based dishes for vegans) than for diet-incongruent dishes (vice versa). Omnivores focused on abstract, long-term consequences of plant-based foods, whereas vegans focused on the socio-political associations with meat foods. Consumption and reward features also positively predicted attractiveness ratings, the likelihood of ordering a dish, and eating intentions. These findings indicate the cognitive processes of polarized dietary groups that may hinder the mainstream transition to more sustainable food choices.

## Introduction

Current mainstream consumption behavior is unsustainable. In particular, global temperatures are estimated to exceed the Paris Agreement 1.5°C target between 2053 and 2061 based on emissions from the food system alone (Clark et al., 2020). Without substantial immediate action, the current trajectory of global warming will likely result in more frequent and extreme weather events, a dramatic loss of biodiversity, and increased poverty worldwide (Hoegh-Guldberg et al., 2019). Moving away from carbon-intense diets, such as those high in meat and dairy, is crucial for minimizing greenhouse gas emissions in high-income countries (Intergovernmental Panel on Climate Change, 2019; Macdiarmid, 2021). However, despite levels of climate crisis awareness being at over 90% (Lee et al., 2015), and public agreement that plant-based diets are good for the environment (Bryant, 2019), mainstream consumers are reluctant to change their eating behavior for environmental reasons (Sanchez-Sabate & Sabaté, 2019). In fact, only one in six omnivores intend to reduce their meat intake (Bryant, 2019).

Social psychology research has a key role to play in the reduction of meat consumption. Indeed, increasing evidence shows that vegans who exclusively consume plant-based foods are seen as a minority group and experience stigma because of their diets (MacInnis & Hodson, 2015; Markowski & Roxburgh, 2019; Wehbe et al., 2023). In addition, omnivores have been found to derogate vegans (De Groeve et al., 2022) for displaying environmentally-friendly dietary behaviors. This suggests polarization between the typical omnivorous consumer and those who follow plant-based diets, which may hinder the mainstream transition to more sustainable lifestyles. This paper therefore examines whether polarization is reflected in how omnivores and vegans cognitively represent (i.e., think about) meat and plant-based foods, as this may influence their motivation to consume them, and can help us better understand the psychological processes underlying the reluctance in dietary change among mainstream consumers.

Why are cognitive representations important? According to the grounded cognition theory of desire (Papies & Barsalou, 2015; Papies, Barsalou, & Rusz, 2020a), when people think about foods, they non-consciously simulate (or re-experience) what it is like to eat them, and if the simulation is rewarding, this can increase desire for those foods. These cognitive, multi-modal simulations are partial re-enactments, which draw upon memories of earlier appetitive experiences and convey vivid sensory detail affecting neural, physiological, and behavioral responses (Krishna & Schwarz, 2014). For example, the same brain regions within the “core eating network,” linked to the sensory processing of taste and reward (Kaye et al., 2009), are activated when presented with a visual food cue as when eating (Chen et al., 2016). Furthermore, asking participants to simulate eating a food increases salivation and desire (Keesman et al., 2016; Muñoz-Vilches et al., 2020). Similarly, situations that are congruent with consuming a food, such as a cinema for popcorn, can cue simulations of eating that food, which increases expected liking and desire (Papies, van Stekelenburg, et al., 2022). Thus, cognitively representing foods in terms of rewarding consumption may be important for understanding the transition to sustainable diets, because the expected enjoyment from eating meat is a key barrier to reducing consumption (Corrin & Papadopoulos, 2017).

Previous research has found that people use a higher proportion of words referring to sensory (e.g., “crunchy”), hedonic (e.g., “tasty”), and contextual (e.g., “summer”) features (i.e., “consumption and reward” features) when describing more attractive foods and drinks, while less attractive foods are described with greater reference to visual aspects (e.g., “red”), ingredients (e.g., “potato”), and the long-term consequences (e.g., “unhealthy”) of consumption (i.e., “situation independent” features; Keesman et al., 2018; Papies, 2013; Papies, Claassen, et al., 2022). In addition, more consumption and reward features are used for more frequently consumed foods and drinks and also predict the desire to consume as well as actual intake in laboratory settings (Papies, Claassen, et al., 2022). Research examining how meat and plant-based foods are presented on social media has also shown that meat foods, which are part of the culturally accepted diet for the majority of Western consumers, are tagged with more words reflecting rewarding eating experiences than plant-based foods (Davis & Papies, 2022). This suggests that attractive and frequently consumed foods are represented through simulations, or re-experiences, of the taste, texture, context, and enjoyment of eating. These simulations reflect previous eating experiences, which shape desire, eating intentions, and actual food choices (Higgs, 2016).

This paper adds to the current literature by examining how omnivores (i.e., whose diet includes animal products) and vegans (i.e., whose diet excludes all animal products) cognitively represent meat and plant-based foods, and how these representations relate to consumption motivation and actual behavior. Using a feature listing task to capture the richness of cognitive representations via natural language descriptors, we asked participants to list the features of a given dish and then analyzed the language used (Wu & Barsalou, 2009). Experiment 1 assessed representations and examined how these representations are associated with ratings of attractiveness and eating motivation judgments. Experiment 2 aimed to replicate the findings from Experiment 1 with a larger sample and also addressed how representations predict consumption intentions and actual consumption over a 30-day follow-up period.

## Experiment 1

We assessed how omnivores and vegans represent diet-congruent dishes (meat dishes for omnivores and plant-based dishes for vegans) and diet-incongruent dishes (plant-based dishes for omnivores and meat dishes for vegans). Although eating plant-based foods is not incompatible with omnivorousness, in practice, omnivores tend to follow meat-centric or meat-rich diets (Michel et al., 2021), and the consumption of specifically plant-based dishes, like those used in the current experiment, is typically infrequent (Dagevos, 2021). Therefore, for simplicity, we refer to plant-based foods as diet-incongruent for omnivores and to meat foods as diet-incongruent for vegans.

Considering previous research has shown that frequently consumed foods are represented in terms of reward (Papies, Claassen, et al., 2022), we hypothesized that omnivores would use more consumption and reward features for meat foods than for plant-based foods (H1) and more situation-independent features for plant-based foods than meat foods (H2). Given that vegans’ dietary choices are motivated by ethical, environmental, and health concerns (Ghaffari et al., 2022), we also hypothesized that vegans would use more situation-independent features than consumption and reward features for both types of food (H3) and would use more situation-independent features than omnivores overall (H4). With regard to the relationship between food representations and desire (Papies, Barsalou, & Rusz, 2020), we also hypothesized that across foods and groups, listing more consumption and reward features would be associated with finding a food more attractive (H5).

We also assessed general eating motivations and how these relate to representations of foods. We hypothesized that, across groups, using more consumption and reward features would be associated with higher scores on the Liking, Pleasure, Affect Regulation, and Need and Hunger subscales (H6a), and that omnivores would score higher on these subscales (H6b). Finally, we hypothesized that, across groups, using more situation-independent words would be associated with higher scores on the Health and Ethical Motivation subscales (H7a), and that vegans would score higher on these subscales (H7b).

### Methods

#### Design and Sample Size

The experiment had a mixed 2 × 2 design to investigate features listed by 2 groups (omnivores and vegans) in response to 2 sets of stimuli (meat and plant-based food dishes). The dependent variables were consumption and reward features, situation-independent features, and dish attractiveness ratings. All variables, measures, and exclusions are reported, and sample sizes were determined before data analysis. Both Experiments 1 and 2 were pre-registered, with all materials available here: https://osf.io/m2t4q/. All pre-registered analyses are reported, along with any deviations from the pre-registered analysis plan.

To determine our sample size, we used G*Power (v3.1; Faul et al., 2009). Our group proportion parameters were set at 0.24 and 0.34, based on the meat (*M* = 0.34) and plant-based (*M* = 0.17) consumption situation means from Davis and Papies (2022), but accounting for a 10% rather than a 17% difference. To find a 10% difference in proportions with a minimum of 80% power at the adjusted .01 alpha, we needed a minimum of 213 participants, or 107 per group. To control for potential exclusions and missing data, we aimed to recruit an additional 5% of participants, totaling 224 participants.

#### Participants

Participants were recruited through Prolific (www.prolific.co). We used custom pre-screening to select eligible participants, who had to confirm that they were: (1) over 18 years old, (2) living in the United Kingdom, (3) fluent in English, and (4) either had no dietary restrictions (omnivore) or followed a vegan diet. Notably, 231 participants completed our experiment, and 11 participants were excluded: five failed attention checks, one gave insufficient responses for the feature listing task, and five gave inconsistent dietary information. Due to a screening error, 38 participants were not currently residing in the United Kingdom (*N_omnivore_* = 7; *N_vegan_* = 31).

Our final sample consisted of 220 participants, which included 109 omnivores (52% female, *M_age_* = 34, *SD_age_* = .27) and 111 vegans (61% female, *M_age_* = 31, *SD_age_* = 10.26), exceeding our planned sample size. All participants received £2.50 for their participation.

#### Materials

Unless otherwise specified, apart from the feature listing task, all responses were given on a 100-point visual analog slider (VAS) scale.

##### Current State

We asked participants to report their current level of hunger and thirst (0 = “*not at all*,” 50 = “*somewhat*,” and 100 = “*extremely*”) separately.

##### Feature Listing Task

All participants were presented with the same 20 dishes: 10 plant-based and 10 meat, matched on dish category (see Table 1). Each dish was presented as follows: “*how would you describe this dish right now?*,” and participants were asked to list at least five features (open text entry). The order of dishes was randomized for each participant.

**Table 1.** List of Dishes.

Dish category	Meat dish	Plant-based dish
Burger	Beef burger	Falafel burger
Pizza	Pepperoni pizza	Vegan pizza
Curry	Chicken tikka masala	Lentil daal
Roast	Roast lamb	Nut roast
Pasta	Beef lasagne	Vegan lasagne
Salad	Chicken caesar salad	Mixed vegetable salad
Fajitas	Chicken fajitas	Vegetable fajitas
Ramen	Pork Ramen	Tofu Ramen
Tagine	Lamb tagine	Chickpea tagine
Steak	Sirloin steak	Cauliflower steak

*Note.* Dishes were chosen by the authors to represent a range of cuisines, ingredients, and categories.

##### Dish Attractiveness

We asked participants “please rate how attractive each meal sounds to you” for each of the 20 dishes (0 = “*not attractive*,” 50 = “*somewhat attractive*,” and 100 = “*very attractive*”).

##### Dish Experience

We asked participants “*please tell us whether you have tried each meal before*” *for each of the 20 dishes, measured on a 3-point scale* (*0* = “*no*,” 1 = “*yes, once*,” and 2 = “*yes, multiple times*”).

##### Eating Motivations

We used the brief version of The Eating Motivation Survey (TEMS; Renner et al., 2012) to measure general eating motivations among participants with different dietary patterns. The brief TEMS is a 45-item questionnaire that covers 15 different food motivations, including Liking, Need and Hunger, Pleasure, Affect Regulation, Sociability, Habits, Health, Visual Appeal, Natural Concerns, Price, Social Norms, Social Image, Traditional Eating, Convenience, and Weight Control. Participants were asked “*I eat what I eat*. . .” and then presented with the scale items (e.g., “. . .*because it tastes good*” and “. . .*because it is inexpensive*”), measured on a 7-point scale (1 = “*never*” and 7 = “*always*”).

We added an Ethical Motivation dimension to the brief TEMS to account for motivations of particular importance among those who follow a plant-based diet, consisting of three items from the Food Choice Questionnaire (Onwezen et al., 2019) as follows: “. . .*because it is environmentally friendly*,” “. . .*because it is animal friendly*,” and “. . .*because it is fairly traded*.” The three items showed good internal consistency (α = .80). Therefore, our final scale consisted of 48 items.

##### Dietary Information

We asked participants to define what dietary group best describes their diet and how long they had followed this diet, measured on a 4-point scale (“*within the last year*,” ‘*within the last 5 years* “*within the last 10 years*,” and “*I’ve always followed this diet*”). We assessed meat consumption frequency by asking participants “*in a typical week, on how many days do you eat meat?*,” measured on an 8-point scale (0 = “*none*” and 7 = “*everyday*”), and “*on a typical day that you eat meat, during how many meals do you eat meat?*,” measured on a 4-point scale (0 = “*I never eat meat*,” and 3 = “*every meal*”). Omnivore participants included those that defined themselves as omnivores, meat and/or dairy reducers, or flexitarians who reported consuming meat at least once per day and once per week. Vegan participants included those that defined themselves as vegans, who reported never consuming meat on the daily and weekly meat consumption frequency measures. Participants who did not fulfill these criteria were excluded from analysis.

##### Demographics

We collected demographic information, including their age (*M* = 32.55, *SD* = 11.37), gender (57% female), nationality (80% U.K. nationals), first language (79% English), country of residence (83% U.K. residents), and subjective socio-economic status (SES), using the MacArthur Scale of Subjective Social Status (Adler & Stewart, 2007) on a 10-point scale (*1* = lowest SES, *10* = highest SES; *M* = 5.59, *SD* = 1.64).

#### Procedure

Data were collected via the Qualtrics software (https://www.qualtrics.com) between 12:00 and 19:00 on December 4, 2020. Participants were required to read the experiment information form and give informed consent before participation. Participants first responded to the current state measures and then completed the feature listing task, after being given detailed instructions. We then asked participants to rate dish attractiveness and dish experience for each of the 20 dishes. Following this, participants completed the eating motivation items and recorded their dietary and demographic information. Participants were fully debriefed at the end of the survey, which took 24 minutes on average to complete.

#### Data Coding

Feature listing responses were coded using the Feature Listing Manual (Papies, Tatar, et al., 2020). Consumption situation features correspond to any immediate or proximal aspect of the situation in which the food is consumed, including the subcategories of sensory and action features (e.g., “creamy,” “crispy,” and “cold”), internal or external context (e.g., “summer,” “restaurant,” and “hungry”), and any immediate positive or negative consequences experienced at the time of consumption (e.g., “tasty,” “disgusting,” and “satisfied”). Situation-independent features include distal or analytical aspects of a food that extend beyond the present consumption situation, such as the ingredients or content of the product (e.g., “tomatoes,” “carbohydrates,” and “dairy-free”), general valence expressions (e.g., “great,” “terrible,” and “awful”), food categories (e.g., “burger,” “fast food,” and “Quorn”), and long-term health consequences (e.g., “healthy,” “fattening,” and “good for your health”). Visual features (e.g., “red,” “cube,” and “layer”) are also included in the situation-independent category, as they can be experienced externally to a particular consumption situation. Non-consumption situation features refer to any aspect of a situation where the food is present but not yet consumed, including the purchase (e.g., “expensive,” “not available,” and “on a budget”), production (e.g., “free range,” “processed,” and “slow cooked”), and preparation (“freeze,” “leftovers,” and “microwave”) of the product. Ambiguous features, that is, those that could be coded into two or more subcategories (e.g., “tea,” “dish,” and “wicked”), and non-word features, such as syncategorematic words that could not be identified as a food word (e.g., “this,” “very,” and “know”), in the experiment language (i.e., English) were coded in a separate category.

We decided to add a social and political context subcategory within the main situation-independent category, to capture the many listed features that referred to general social norms or political references. This largely consisted of features surrounding the ethics of a dish (e.g., “animal abuse,” “bad for the environment,” and “unjust”), the production of the dish that holds emotional or explicit imagery (e.g., “carcass,” “born to die,” and “pigs screaming”), and social-political discourse relating to dietary practices (e.g., “stop killing animals,” “why kill for pleasure,” and “people are barbaric”). These responses seemed important to code separately, as they communicate attitudes relating to the intersection between (vegan) identity and food representations. We also added the inexperience subcategory to the non-word category, to capture any features relating to not knowing or having an experience of a dish (e.g., “clueless,” “never tried,” and “unfamiliar”). Therefore, 5 main categories and 44 subcategories in total were used during coding.

Features that consisted of several words were divided into the smallest meaningful units and coded separately. For example, “dinner with friends” became “dinner” (consumption situation: time setting and frequency) and “with friends” (consumption situation: social setting). For more details on the coding procedure and associated ShinyApp, see Papies, Tatar, et al. (2020).

LH coded all features, and secondary coding was completed by TD to test for interrater reliability (McHugh, 2012). The secondary coding sample size was 10% of the total unique words coded. Results from secondary coding a randomized sample of 427 words showed moderate agreement (κ = 0.69, % agreement = 0.79) at the main category level on which our hypotheses focused, which was deemed adequate for our analyses.

#### Analysis Plan

We calculated the key dependent variable of consumption and reward features as the proportion of sensory and action features, context features, and immediate positive consequence features, divided by all features coded across the three main categories (consumption situation, situation-independent, and non-consumption situation features). The proportion of situation-independent features was calculated by dividing the number of situation-independent features by that same total. For example, if a participant used three situation-independent features out of five coded features total when describing a dish, the situation-independent proportion for that response would be 0.60. Ambiguous and non-word categories were excluded from the analysis, as these were considered separate from the food language of interest.

All analyses were conducted in R (version 4.2.2; R Core Team, 2022), with data cleaning and visualization processed using the *tidyverse* library and associated packages (version 1.3.2; Wickham et al., 2019), except for our raincloud plots (Allen et al., 2019) and wordclouds (version 2.6; Fellows, 2018). For H1 to H5 and H6a and H7a models, we fitted linear mixed-effects models with the *lmer* function of the *lme4* package (version 1.1-31; Bates et al., 2015). For H6b and H7b, we ran independent samples *t*-tests. Across all confirmatory models, we employed a maximal random-effects structure (Barr et al., 2013). For our exploratory H1 (vegan) model and H3 model, we included random intercepts and slopes for each participant and dish. For H1, H2, H5 (vegan), and exploratory social and political context models, we included random intercepts and slopes for each participant and random intercepts for each dish. For H4, H5 (omnivore), H6a, H7a, and exploratory demographics models, we included random intercepts only for each participant and dish.

We predicted proportions (logit transformed) with a fixed effect of diet for our H1 to H4 models and a fixed effect of TEMS subscales (standardized) for our H6a and H7a models. For our H5 model, dish attractiveness (standardized) was predicted with a fixed effect of consumption and reward proportions (logit transformed). We decided to measure omnivore and vegan responses separately for the H5, H6a, and H7a models and focused on diet-congruent dishes only. As such, omnivore and vegan responses were rescaled in separate datasets. Given the low consumption frequency of diet-incongruent dishes, especially among vegans, measuring the relationship between consumption and reward features and the consumption behavior of dishes that aligned with participants’ dietary preferences seemed more suitable for the interpretation of our data. Nevertheless, omnivore results for the confirmatory models including diet-incongruent dishes can be found in the Supplementary Online Materials (SOM).

To control for familywise error rate from multiple testing across H1 to H5, we adjusted our alpha level in Experiment 1 to α = .01 using the Bonferroni correction (.05/5 = .01). Model diagnostics were assessed using the *DHARMa* package (version 0.4.6; Hartig, 2022). Our models showed small deviations from the expected distribution (Kolmogorov–Smirnov test). However, these results are unlikely to influence type 1 error rate, standard error, or empirical power estimates, and therefore we decided to run the models without corrections.

Finally, we obtained an estimate of variance explained with the *r.squaredGLMM* function from the *MuMIn* package (version 1.47.1; Bartoń, 2022). Marginal and conditional R-squared coefficients were calculated, with marginal R-squared (*R*^2^_*m*_) representing the variance explained by just the fixed effects and conditional R-squared (*R*^2^_*c*_) representing the variance explained by the entire model, including both fixed and random effects (Nakagawa & Schielzeth, 2013).

### Results

A total of 23,869 features, or 3,910 (16%) unique, were coded. Omnivores generated 11,552, or 1,283 unique (11%), features, whereas vegans generated 12,317, or 1,729 unique (14%), features. Omnivores (*M* = 5.30, *SD* = 1.32) listed fewer features than vegans (*M* = 5.55, *SD* = 1.01), *t*(4,074.5) = −7.10, *p* < .001, *d* = −0.21. In addition, 12,020 features, or 1,468 unique (12%), were used to describe meat dishes, and 11,849 words, or 1,552 unique (13%), were used to describe plant-based dishes. On average, more features were used to describe meat dishes (*M* = 5.47, *SD* = 1.18) than plant-based dishes (*M* = 5.39, *SD* = 1.18), but this was not significant with the corrected alpha, *t*(4,397) = 2.26, *p* = .02.

**Figure 1.**
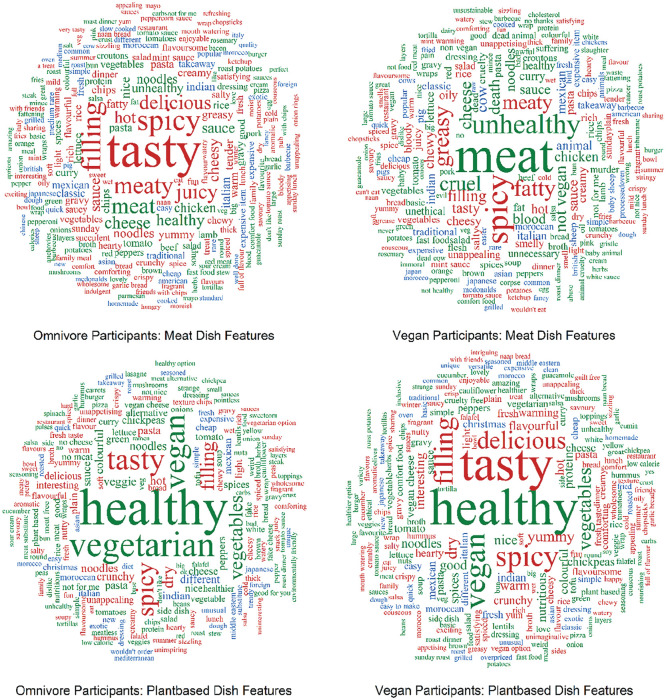
Wordclouds of feature frequencies by diet and dish type across Experiments 1 and 2. *Note.* Situation-independent features are shown in green, consumption and reward features are shown in red, and non-consumption situation features are shown in blue.

Proportion means for the feature listing categories can be found in Table 2, and a visualization of the most popular features by diet and dish type for both experiments can be found in Figure 1. Across both experiments, the highest frequency words among omnivores for meat dishes were “tasty” (*N* = 1,546), “spicy” (*N* = 836), and “filling” (*N* = 764), and for plant-based dishes were “healthy” (*N* = 1,537), “vegetarian” (*N* = 996), and “vegan” (*N* = 858). For vegans, the highest frequency words used for meat dishes were “meat” (*N* = 1,133), “spicy” (*N* = 670), and “unhealthy” (*N* = 544), and for plant-based dishes were “healthy” (*N* = 1,343), “tasty” (*N* = 1,306), and “vegan” (*N* = 755).

**Table 2.** Experiment 1 Feature Listing Category Means and Standard Deviations by Diet and Dish Type.

Diet	Dish type	Consumption and reward		Situation independent		Non-consumption situation	
		*M*	*SD*	*M*	*SD*	*M*	*SD*
Omnivore							
	Meat	0.45	0.27	0.37	0.26	0.16	0.18
	Plant-based	0.28	0.24	0.49	0.26	0.13	0.16
	Total	0.37	0.27	0.43	0.27	0.14	0.17
Vegan							
	Meat	0.25	0.23	0.48	0.25	0.17	0.18
	Plant-based	0.37	0.24	0.44	0.25	0.15	0.16
	Total	0.31	0.24	0.46	0.25	0.16	0.17

Overall, participants had not tried 6 out of 20 dishes (*M* = 6.33, *SD* = 3.16). Both omnivores (*M* = 5.17, *SD* = 2.30) and vegans (*M* = 4.73, *SD* = 2.96) had not tried 5 diet-incongruent dishes and 2 diet-congruent dishes (*M_Omnivore_* = 1.90, *SD_Omnivore_* = 1.08; *M_Vegan_* = 2.32, *SD_Vegan_* = 1.40). For further descriptives, see the SOM on the project OSF page.

#### Confirmatory Analyses

##### Consumption and Reward Features (H1)

**Figure 2.**
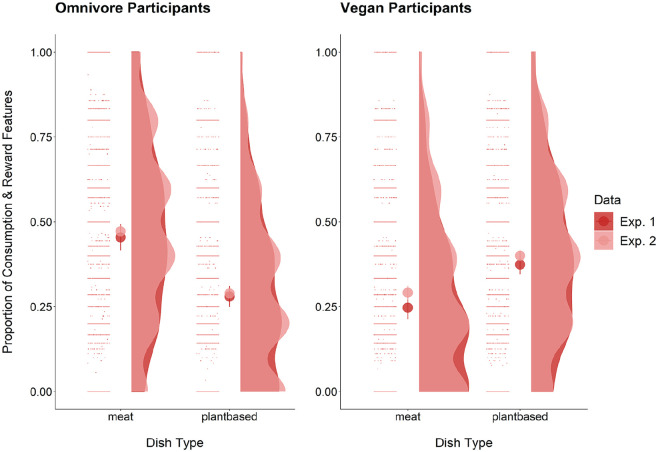
Raincloud plot of Experiment 1 and 2 mean values of consumption and reward features by diet and dish type. *Note*. The scatterplot and violin plot elements represent the distribution of the proportions for all observations, whereas the middle points represent the average proportion means.

In line with our hypothesis, omnivores listed more consumption and reward features for meat dishes than plant-based dishes, *b* = 0.55, *SE* = 0.10, *p* < .001, *R*^2^_*m*_ = 0.10, *R*^2^_*c*_ = 0.45 (see Figure 2). Exploratory analyses revealed the reverse effect among vegans, who used fewer consumption and reward features for meat dishes than plant-based dishes, *b* = −0.41, *SE* = 0.08, *p* < .001, *R*^2^_*m*_ = 0.07, *R*^2^_*c*_ = 0.37.

##### Situation-Independent Features (H2–H4)

**Figure 3.**
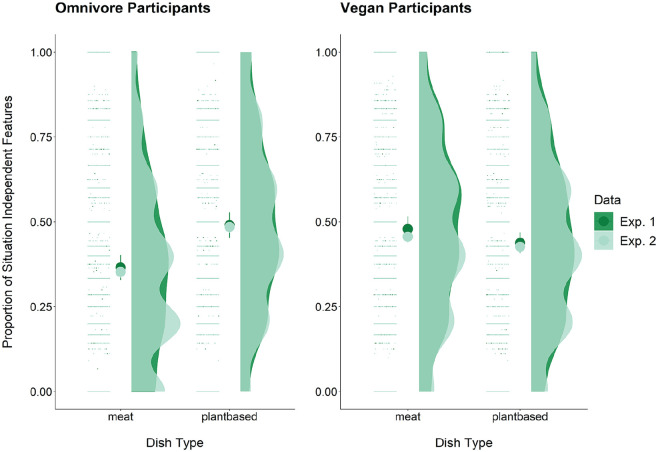
Raincloud plot of Experiment 1 and 2 situation-independent means by diet and dish type.

As predicted in Hypothesis 2, omnivores used fewer situation-independent features for meat dishes than plant-based dishes, *b* = −0.38, *SE* = 0.10, *p* = .002, *R*^2^_*m*_ = 0.05, *R*^2^_*c*_ = 0.45 (see Figure 3). In addition, in line with Hypothesis 3, vegans used fewer consumption and reward features than situation-independent features in general, *b* = −0.45, *SE* = 0.11, *p* < .001, *R*^2^_*m*_ = 0.08, *R*^2^_*c*_ = 0.35. We also hypothesized that vegans would use more situation-independent features than omnivores overall (H4). Contrary to our predictions, there was no difference in the use of situation-independent words between vegans and omnivores, *b* = −0.08, *SE* = 0.06, *p* = .17.

##### Dish Attractiveness (H5)

As hypothesized, for diet-congruent dishes, listing more consumption and reward features was associated with higher attractiveness ratings among omnivores, *β* = 0.11, *SE* = 0.02, *p* < .001, *R*^2^_*m*_ = 0.03, *R*^2^_*c*_ = 0.20, and among vegans, *β* = 0.17, *SE* = 0.02, *p* < .001, *R*^2^_*m*_ = 0.06, *R*^2^_*c*_ = 0.35.

##### Eating Motivations

We predicted that using more consumption and reward features would be associated with higher scores on the TEMS Liking, Affect Regulation, Need and Hunger, and Pleasure subscales (H6a). However, contrary to our predictions, there was no association between diet-congruent consumption and reward features and these subscales for omnivores or vegans (see Table 3). In line with H6b, omnivores scored higher than vegans on Pleasure, but not on Liking, Need and Hunger, or Affect Regulation (see Table 4). We also hypothesized that using more situation-independent features would be associated with higher TEMS Health and FIQ Ethical Motivation scores (H7a). However, there was no association between these subscales and situation-independent features for diet-congruent foods among omnivores or vegans (see Table 3). In line with H7b, omnivores scored lower than vegans on Health and Ethical Motivation (see Table 4). Exploratory analyses showed that, when including the TEMS subscales as covariates within the other confirmatory models, these subscales did not display any significant effects (see the SOM for further details).

**Table 3.** Means, Standard Deviations, and Model Statistics for Eating Motivations Predicting Feature Listing Category Proportions.

	Omnivore		Vegan	
	*M* (*SD*)	*b* (*SE*) *p*	*M* (*SD*)	*b* (*SE*) *p*
Consumption and reward				
Liking	6.06 (0.75)	0.14 (0.12) .25	6.02 (0.67)	0.05 (0.09) .59
Affect Regulation	2.67 (1.42)	−0.14 (0.11) .21	2.53 (1.34)	−0.07 (0.09) .45
Need and Hunger	5.34 (0.82)	−0.06 (0.11) .59	5.39 (0.79)	−0.09 (0.09) .30
Pleasure	5.02 (0.92)	0.12 (0.13) .35	4.67 (0.96)	0.13 (0.09) .17
Situation independent				
Health	4.31 (1.27)	−0.18 (0.11) .11	4.84 (1.06)	<0.01 (0.09) .99
Ethical Motivation	3.21 (1.13)	−0.02 (0.16) .92	5.58 (0.92)	0.05 (0.09) .57

*Note. SE* = standard error.

**Table 4.** Means and *t*-Test Results for Eating Motivation Differences Between Omnivores and Vegans.

Motivation	Omnivore		Vegan		*t*	*df*	*p*	*d*
	*M*	*SD*	*M*	*SD*				
Ethical Motivation	3.21	1.14	5.58	0.92	–16.94	207.59	<.001	–2.29
Traditional Eating	4.22	1.05	2.88	1.26	9.24	218	<.001	1.25
Sociability	3.83	1.31	2.52	1.33	7.39	218	<.001	0.98
Social Norms	3.30	1.16	2.35	1.20	6.02	218	<.001	0.81
Natural Concerns	3.48	1.38	4.30	1.45	–4.28	218	<.001	–0.58
Social Image	2.38	0.96	1.84	0.93	4.18	218	<.001	0.56
Health	4.31	1.27	4.84	1.07	–3.35	210.41	<.001	–0.45
Habits	5.06	0.90	4.64	1.09	3.10	218	.002	0.42
Pleasure	5.02	0.92	4.67	0.97	2.81	218	.005	0.38
Visual Appeal	3.60	1.06	3.20	1.10	2.70	218	.007	0.37
Price	4.12	1.07	3.85	1.23	1.72	218	.09	0.23
Convenience	4.64	0.88	4.45	1.06	1.41	218	.16	0.19
Affect Regulation	2.67	1.43	2.53	1.35	0.75	218	.45	0.10
Need and Hunger	5.34	0.83	5.39	0.79	–0.41	218	.68	–0.06
Liking	6.06	0.75	6.02	0.67	0.39	218	.70	0.05
Weight Control	3.29	1.16	3.30	1.38	–0.04	212.69	.97	–0.01

#### Exploratory Analyses

We explored the effects of diet and dish type on the novel social and political context subcategory, which we had added to our coding manual to accommodate uncategorized language in our dataset. A total of 793 social and political context features, 283 unique (36%), were coded, with the majority being used by vegan participants (95.33%) to describe meat dishes (87.77%). Notably, 70% of vegan participants (*N* = 78) used at least one social and political context feature, in contrast to 24% (*N* = 26) of omnivores. In addition, we found that 10 vegan participants contributed to 41% (*N = 322*) of all social and political context features. The most popular social and political context feature used was “cruel” (*N* = 69), followed by “death” (*N* = 43) and “cruelty” (*N* = 29). We ran a binomial mixed-effects model among vegans to determine the effect of dish type on social and political context features. Vegans used more social and political context features for meat dishes (*M* = 0.12, *SD* = 0.20) than plant-based dishes (*M* = 0.01, *SD* = 0.05), *b* = 0.46, *SE* = 0.07, *p* < .001, *R*^2^_*m*_ = 0.14, *R*^2^_*c*_ = 0.66.

### Discussion

Participants described diet-congruent foods with more consumption and reward features than diet-incongruent foods, and these features were positively associated with finding a diet-congruent dish attractive. In other words, “ingroup” foods were represented more in terms of the pleasure of eating than “outgroup” foods, reflecting dietary polarization. Among omnivores, diet-incongruent foods were described with more features independent of the consumption situation than diet-congruent foods. Vegans used more of these features overall, especially social and political context features for meat foods. Although omnivores were more driven by pleasure and affect eating motives, and vegans by health and ethical eating motives, there was no strong pattern of associations between features used and self-reported eating motivations, as assessed by established eating motivation scales. To provide further insights into the consumption associations of these cognitive representations, we measured the association of food representations with behavior over time in Experiment 2.

## Experiment 2

This study was designed to replicate findings from Experiment 1 with a larger sample and extend our understanding of the relationship between consumption and reward features and behavioral outcomes. Hence, we added measures of typical consumption and consumption intentions and actual consumption over a 30-day follow-up period. While previous research has shown that consumption and reward features predict intake in a laboratory setting (Papies, Claassen, et al., 2022), no work so far has examined whether they predict intake outside the laboratory and over time. We were particularly interested to see whether simulations of consuming and enjoying a dish would predict consumption over and above typical consumption frequency, as this might reflect an effect of desire arising from consumption and reward simulations. In other words, past behavior has consistently been found to predict both intentions and prospective behavior (Dean et al., 2012; McEachan et al., 2011), which is consistent with research exploring the strong role of habits within eating behavior (see Riet et al., 2011). Here, we were interested in the unique effects of cognitively representing a food in terms of consumption and reward on both intentions and behavior, even when controlling for typical, habitual consumption.

We again hypothesized that participants would use a higher proportion of consumption and reward features for diet-congruent dishes than diet-incongruent dishes (H1). We further predicted that participants would use a higher proportion of situation-independent features to describe diet-incongruent dishes than diet-congruent dishes (H2). We also hypothesized that across groups, listing more consumption and reward features for a dish would predict the likelihood of ordering that dish (H4).

To assess whether consumption and reward simulations predict behavioral outcomes in a real-world context, we hypothesized that across groups, the proportion of consumption and reward features would predict both consumption intentions (H6a) and actual consumption (H5a), when controlling for how often participants typically consume a dish (H5b, H6b), which we expected to predict consumption and reward features separately (H3). In essence, when controlling for the effect of typical consumption, consumption and reward features would positively predict consumption intentions and actual consumption frequency at follow-up.

### Methods

#### Design and Sample Size

The experiment again had a mixed 2 (omnivore, vegan) × 2 (meat dish, plant-based dish) design. Diet, dish type, and typical consumption were the independent variables. Consumption and reward and situation-independent features, ordering likelihood, consumption intentions, and actual consumption were the dependent variables.

We conducted a power analysis using G*Power. Our analysis was based on a generic binomial test, with group proportion parameters set at 0.45 and 0.50, based on the smallest proportional difference found in Experiment 1 between meat dish (*M* = 0.48) and plant-based dish (*M* = 0.44) situation-independent features among vegan participants, but accounting for a 5% difference instead of a 4% difference. To detect a 5% difference in proportions between groups, with a minimum of 80% power and an adjusted alpha of 0.025, we need a minimum of 786 participants, or 393 per group. To account for potential exclusions, missing data, and attrition, we recruited an extra 8% of participants (848 participants total).

#### Participants

Participants were again recruited through Prolific. We specified the same inclusion criteria as Experiment 1 and used custom pre-screening to select eligible participants, which excluded those who had taken part in Experiment 1. A total of 911 participants completed our Time 1 questionnaire, and 74% of these participants completed at Time 2. Notably, 68 participants were excluded; 8 failed attention checks, 12 gave insufficient responses to the feature listing task, and 48 gave inconsistent dietary information.

The final sample consisted of 843 participants, including 436 omnivores (71% female, *M_age_* = 33.88, *SD_age_* = 11.86) and 407 vegans (75% female, *M_age_* = 32.21, *SD_age_* = 10.77), exceeding our planned sample size. Of these, 674 participants, including 351 omnivores and 323 vegans, had Time 2 data. Participants received £1.75 for their participation at Time 1 and £0.33 for their participation at Time 2.

#### Materials

Unless otherwise specified, all responses were given on a 100-point VAS scale.

##### Current State

We measured hunger and thirst the same as in Experiment 1.

##### Feature Listing Task

This was the same as in Experiment 1, with the addition that participants were prompted to imagine that they were in a restaurant setting, presented with a menu visualization with the dish name included. Examples of the menu visualizations can be found in the SOM.

##### Ordering Likelihood

We asked participants “*how likely is it that you would order* [DISH NAME] *from the menu?” for each of the 20 dishes (0 = “very unlikely*,” 100 = “*very likely*”).

##### Consumption Behavior

We measured consumption behavior for each of the 20 dishes. Typical consumption responses were collected at Time 1 by asking “*typically, how often do you consume the following dishes?” (0 = “never*” and 100 = “*very often*”). Consumption intentions were also measured at Time 1, where participants were asked “*to what extent do you agree with the following statement for each food below: I intend to consume this food in the next month” (0 = “strongly disagree*” and 100 = “*strongly agree*”). At Time 2, we collected actual consumption responses, with the question “*in the past month, how often have you consumed the following dishes?” (0 = “never*” and 100 = “*very often*”).

##### Dietary Information

We collected the same dietary information as in Experiment 1. However, we added the response “*within the last 3 years” to the diet length question and also gathered information on control over household food decisions, by asking “to what degree do you decide what is consumed as the main meals in your household?” (0 = “I never decide*” and 100 = “*I always decide*”).

##### Demographics

We collected the same demographic information as in Experiment 1, including age (*M* = 33.07, *SD* = 11.39), gender (73% female), nationality (91% U.K. nationals), first language (95% English), and subjective SES (*M* = 5.30, *SD* = 1.66).

#### Procedure

Time 1 data were collected via Qualtrics from midday between September 6, 2021, and September 28, 2021, and Time 2 data from midday between October 7, 2021, and November 5, 2021. Participants were required to read the experiment information form and give informed consent before participation in both surveys.

At Time 1, participants first responded to the current state measures and completed the feature listing task after being given detailed instructions. Participants then completed the ordering likelihood measure for each of the 20 dishes. After this, participants completed the typical consumption, consumption intentions and both dietary information and demographic measures. At Time 2, between 30 and 40 days later, participants were asked to complete the actual consumption measure, and indicated if their dietary behavior had changed. Participants were fully debriefed at the end of each survey. Time 1 took 22 minutes on average to complete, and Time 2 took 3 minutes on average.

#### Data Coding

Participant responses were coded using the same coding procedure and categories as in Experiment 1. TD coded all features.

#### Analysis Plan

Analyses followed the same procedures as in Experiment 1. We fitted linear mixed-effects models using the *lmer* function of the *lme4* package. Models for H3, H4, H6a (omnivore), and exploratory consumption intentions (omnivore) included random intercepts and slopes for each participant and dish. Models for H1, H2 (omnivore), H5a, H6a (vegan), H6b (vegan), exploratory social and political context features, and exploratory consumption intentions (vegan) included random intercepts and slopes for each participant and random intercepts for each dish. Models for H2 (vegan), H5b, H6b (omnivore), and exploratory demographics included random intercepts only for each participant and dish.

We predicted proportions (logit transformed) with a fixed effect of diet for H1 and H2, and a fixed effect of typical consumption (standardized) for H3. We predicted ordering likelihood (standardized) with a fixed effect of consumption and reward proportions (standardized) for H4. We also predicted actual consumption (standardized) for H5, and consumption intentions (standardized) for H6, with fixed effects for typical consumption (standardized) and consumption and reward proportions (logit transformed). We again decided to run separate models for omnivores and vegans throughout our analysis and focused on diet-congruent dishes only for H3 to H6. For omnivore model results including diet-incongruent dishes, see the SOM.

To control for familywise error rate from multiple testing across H1 and H2, we adjusted our alpha level in Experiment 1 to α = .025 using the Bonferroni correction (.05/2 = .025). Model diagnostics for all models showed small deviations from the expected distribution (Kolmogorov–Smirnov test) and outlier violations. We decided to run models, like in Experiment 1, without corrections.

### Results

**Figure 4.**
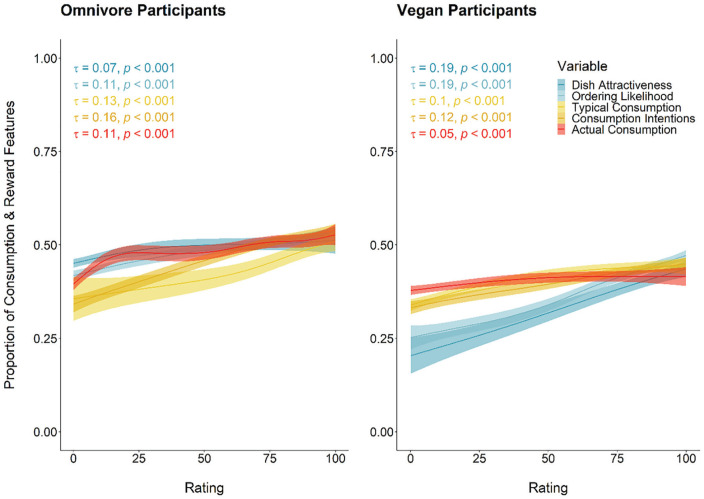
LOESS lines and correlation coefficients (Kendall’s tau) between consumption and reward proportions and key outcome variables in Experiments 1 and 2 for diet-congruent dishes. *Note.* Dish attractiveness was measured in Experiment 1. Ordering likelihood, typical consumption, consumption intentions, and actual consumption were measured in Experiment 2.

A total of 91,363 features, or 7,346 (8%) unique, were coded. Omnivores generated 46,581, or 2,423 unique (5%), features, whereas vegans generated 44,782, or 2,772 unique (6%) features. Like in Experiment 1, omnivores (*M* = 5.34, *SD* = 1.48) reported fewer features than vegans (*M* = 5.50, *SD* = 1.31), *t*(16,803) = −7.39, *p* < .001, *d* = −0.11. In addition, 45,869 features, or 2,769 unique (6%), were used to describe meat dishes, and 45,494 words, or 2,685 unique (6%), were used to describe plant-based dishes. On average, more features were used to describe meat dishes (*M* = 5.44, *SD* = 1.24) than plant-based dishes (*M* = 5.40, *SD* = 1.55), but like in Experiment 1, this was not significant with our corrected alpha, *t*(16,857) = 2.03, *p* = .04. Proportion means for the feature listing categories can be found in Table 5. A visualization of the relationship between consumption and reward features and the behavioral outcome variables across Experiments 1 and 2 can be found in Figure 4.

**Table 5.** Experiment 2 Feature Listing Category Means and Standard Deviations per Diet and Dish Type.

Diet	Dish type	Consumption and reward		Situation independent		Non-consumption situation	
		*M*	*SD*	*M*	*SD*	*M*	*SD*
Omnivore							
	Meat	0.47	0.26	0.35	0.26	0.15	0.17
	Plant-based	0.29	0.24	0.49	0.26	0.11	0.15
	Total	0.38	0.27	0.42	0.27	0.13	0.16
Vegan							
	Meat	0.29	0.25	0.46	0.26	0.16	0.17
	Plant-based	0.40	0.25	0.43	0.24	0.12	0.15
	Total	0.35	0.26	0.44	0.25	0.14	0.16

Overall, participants reported a typical consumption score of 0 for 9 of the 20 dishes presented (*M* = 8.53, *SD* = 4.09). Omnivores did not typically consume 5 diet-incongruent dishes (*M* = 4.92, *SD* = 3.10) and 2 diet-congruent dishes (*M* = 1.77, *SD* = 1.90), whereas vegans did not typically consume 9 diet-incongruent dishes (*M* = 9.31, *SD* = 2.16) and 1 diet-congruent dish (*M* = 1.21, *SD* = 1.59).

#### Confirmatory Analyses

##### Consumption and Reward Features (H1)

As predicted, more consumption and reward features were used by omnivores for meat dishes than plant-based dishes, *b* = 0.54, *SE* = 0.09, *p* < .001, *R*^2^_*m*_ = 0.10, *R*^2^_*c*_ = 0.39, and fewer were used by vegans for meat dishes than plant-based dishes, *b* = −0.35, *SE* = 0.07, *p* < .001, *R*^2^_*m*_ = 0.05, *R*^2^_*c*_ = 0.42 (see Figure 2).

##### Situation-Independent Features (H2)

In line with our predictions, omnivores used fewer situation-independent features for meat dishes than plant-based dishes, *b* = −0.40, *SE* = 0.09, *p* < .001, *R*^2^_*m*_ = 0.06, *R*^2^_*c*_ = 0.42, but vegans did not use more of these features for meat dishes than plant-based dishes, *b* = 0.08, *SE* = 0.06, *p* = .19 (see Figure 3).

##### Ordering Likelihood (H4)

In line with our hypothesis, listing more consumption and reward features for diet-congruent dishes was associated with a higher likelihood of ordering among omnivores, *β* = 0.16, *SE* = 0.02, *p* < .001, *R*^2^_*m*_ = 0.06, *R*^2^_*c*_ = 0.36, and among vegans, *β* = 0.17, *SE* = 0.02, *p* < .001, *R*^2^_*m*_ = 0.06, *R*^2^_*c*_ = 0.37.

##### Consumption Behavior

As hypothesized (H3), the typical consumption frequency of a diet-congruent dish was associated with greater proportion of consumption and reward features used to describe that dish, for omnivores, *β* = 0.27, *SE* = 0.04, *p* < .001, *R*^2^*_m_* = 0.03, *R*^2^_*c*_ = 0.38, and for vegans, *β* = 0.26, *SE* = 0.04, *p* < .001, *R*^2^*_m_* = 0.03, *R*^2^_*c*_ = 0.33.

We hypothesized that the proportion of consumption and reward features would predict actual consumption (H5a), in addition to typical consumption (H5b). We found that consumption and reward features positively predicted actual consumption for diet-congruent dishes, among omnivores, *β* = 0.06, *SE* = 0.01, *p* < .001, *R*^2^*_m_* = 0.01, *R*^2^_*c*_ = 0.48, and among vegans, *β* = 0.06, *SE* = 0.01, *p* < .001, *R*^2^*_m_* = 0.01, *R*^2^_*c*_ = 0.50. However, contrary to our predictions, when controlling for typical consumption, consumption and reward features were not associated with actual consumption for omnivores, *β* = 0.01, *SE* = 0.01, *p* = .64, or for vegans, *β* = 0.01, *SE* = 0.01, *p* = .44. This is likely due to a strong relationship between typical consumption and actual consumption in the model, for omnivores, *β* = 0.51, *SE* = 0.01, *p* < .001, and for vegans, *β* = 0.48, *SE* = 0.01, *p* < .001. The overall model explained a moderate amount of the variation in actual consumption for omnivores, *R*^2^_*m*_ = 0.29, *R*^2^_*c*_ = 0.56, and for vegans, *R*^2^_*m*_ = 0.27, *R*^2^_*c*_ = 0.57.

We hypothesized that the proportion of consumption and reward features would predict consumption intentions (H6a) when controlling for typical consumption (H6b). We found a positive relationship between consumption and reward features and consumption intentions for diet-congruent dishes, among omnivores, *β* = 0.10, *SE* = 0.01, *p* < .001, *R*^2^*_m_* = 0.02, *R*^2^_*c*_ = 0.42, and among vegans, *β* = 0.06, *SE* = 0.02, *p* < .001, *R*^2^*_m_* = 0.01, *R*^2^_*c*_ = 0.50. In line with our predictions, higher consumption and reward features also predicted consumption intentions, *β* = 0.02, *SE* = 0.01, *p* < .001, when controlling for typical consumption frequency, *β* = 0.81, *SE* = 0.01, *p* < .001, for omnivores. We found the same relationship between consumption and reward features and consumption intentions for vegans, *β* = 0.04, *SE* = 0.01, *p* < .001, when controlling for typical consumption, *β* = 0.71, *SE* = 0.01, *p* < .001. The overall model explained a very large amount of the variation in consumption intentions, for omnivores, *R*^2^_*m*_ = 0.68, *R*^2^_*c*_ = 0.75, and for vegans, *R*^2^_*m*_ = 0.55, *R*^2^_*c*_ = 0.71.

#### Exploratory Analyses

##### Social and Political Context Features

We wanted to replicate findings from Experiment 1 exploring the effect of diet and dish type on social and political context features. A total of 2,941 social and political context features, or 572 unique (19.45%), were coded in Experiment 2, again with the majority being used by vegan participants (90.62%) to describe meat dishes (82.31%). Notably, 67% of vegan participants (*N* = 273) used at least one social and political context feature, in contrast to 25% (*N* = 108) of omnivores. In addition, we found that 10 vegan participants contributed to 16% (*N* = 462) of all social and political context features. Like in Experiment 1, the most popular social and political context feature used was “cruel” (*N* = 139), followed by “death” (*N* = 210) and “unethical” (*N* = 108). We ran a binomial mixed-effects model among vegans to determine the effect of dish type on social and political context features. Vegans used more social and political context features for meat dishes (*M* = 0.11, *SD* = 0.19) than plant-based dishes (*M* = 0.01, *SD* = 0.05), *b* = 0.44, *SE* = 0.06, *p* < .001, *R*^2^_*m*_ = 0.13, *R*^2^_*c*_ = 0.67.

##### Consumption Intentions and Actual Consumption

Exploring whether consumption and reward features indirectly predicted consumption of diet-congruent dishes via intentions, we found a positive relationship between consumption intentions and actual consumption, for omnivores, *β* = 0.49, *SE* = 0.04, *p* < .001, *R*^2^*_m_* = 0.27, *R*^2^_*c*_ = 0.62, and for vegans, *β* = 0.45, *SE* = 0.02, *p* < .001, *R*^2^_*m*_ = 0.22, *R*^2^_*c*_ = 0.59. We then ran a model with consumption intentions mediating the relationship between consumption and reward features and actual consumption. Indeed, consumption and reward features indirectly predicted actual consumption through consumption intentions, for omnivores, *β* = 0.06, 95% confidence interval (CI) [0.05, 0.07], *p* < .001, and for vegans, *β* = 0.07, 95% CI [0.06, 0.08], *p* < .001.

### Discussion

Similar to Experiment 1, participants described diet-congruent “ingroup” foods with more consumption and reward features than diet-incongruent “outgroup” foods, and these features were positively associated with the self-reported likelihood of ordering a dish (see Table 6). Again, omnivores described diet-incongruent foods with more features independent of the consumption situation, and vegans with more social and political context features, than diet-congruent foods, reflecting the polarization also observed in Experiment 1. Frequently consuming a diet-congruent dish was positively associated with consumption and reward features. These features predicted intentions to consume a dish, as well as actual consumption (over a 30-day period), but this effect disappeared when controlling for typical consumption frequency. Nonetheless, describing a diet-congruent dish with more consumption and reward features indirectly predicted actual consumption through intentions. This suggests that participants’ intentional and habitual consumption behavior may be closely aligned.

**Table 6.** Summary of Results for Experiment 1 and 2 Confirmatory Hypotheses.

Number	Hypothesis	Supported?
Experiment 1		
H1	Omnivores use more consumption and reward features for meat foods than for plant-based foods	Yes
H2	Omnivores use more situation-independent features for plant-based foods than meat foods	Yes
H3	Vegans use more situation-independent features than consumption and reward features for both types of food	Yes
H4	Vegans use more situation-independent features than omnivores overall	No
H5	Using more consumption and reward features would be associated with higher Dish Attractiveness scores	Yes
H6a	Using more consumption and reward features would be associated with higher scores on the Liking, Pleasure, Affect Regulation, and Need and Hunger subscales	No
H6b	Omnivores score higher on the Liking, Pleasure, Affect Regulation, and Need and Hunger subscales	Partially
H7a	Using more situation-independent words would be associated with higher scores on the Health and Ethical Motivation subscales	No
H7b	Vegans score higher on the Health and Ethical Motivation subscales	Yes
Experiment 2		
H1	Participants use a higher proportion of consumption and reward features for diet-congruent dishes than diet-incongruent dishes	Yes
H2	Participants use a higher proportion of situation-independent features to describe diet-incongruent dishes than diet-congruent dishes	Partially
H3	Greater typical consumption of a dish positively predicts the use of consumption and reward features when describing that dish	Yes
H4	Using more consumption and reward features for a dish positively predicts the likelihood of ordering a dish	Yes
H5a	Using more consumption and reward features positively predicts the actual consumption of a dish	Yes
H5b	Using more consumption and reward features positively predicts the actual consumption of a dish when controlling for how often participants typically consume a dish	No
H6a	Using more consumption and reward features positively predicts the consumption intentions for a dish	Yes
H6b	Using more consumption and reward features positively predicts the consumption intentions for a dish when controlling for how often participants typically consume a dish	Yes

## General Discussion

Across two experiments, we investigated differences in the representation of meat and plant-based foods between omnivores and vegans, and whether these representations predict desire and consumption behavior. In line with our hypotheses, participants consistently used more consumption and reward features to represent diet-congruent “ingroup” foods than diet-incongruent “outgroup” foods. These features, directly associated with typical consumption, were related to the perceived attractiveness and likelihood of ordering a diet-congruent dish, and also predicted consumption intentions (when controlling for typical consumption), which in turn predicted actual consumption. This provides evidence for the relationship between cognitive representations and consumption outcomes for two distinct dietary groups.

Furthermore, we discovered that omnivores use more situation-independent features, and vegans use in particular more social and political context features, for diet-incongruent dishes than diet-congruent dishes. These findings characterize the similarities and differences between these groups in how they think about “outgroup” foods, with a focus on abstract information, rather than on eating experiences and enjoyment. Although there were differences in eating motivations among omnivores and vegans, these were not associated with how diet-congruent foods were described. This suggests that conscious motivations for consumption as measured by self-report scales are not reflected in how foods are represented cognitively when assessed in a free production task, that is, feature listing. It is possible that the general motivation that informs food choices when averaged across foods and situations, as assessed with TEMS, is quite different from what drives preferences and choices with regard to a particular dish in a specific situation, as assessed with the feature listing task. Further research should assess this more directly.

### Applied Implications

Our results correspond with previous findings that meat and plant-based foods are presented differently in both real-world and online contexts. Within supermarkets and on social media, meat foods have been found to be presented with more consumption and reward language than plant-based foods, while plant-based foods were described with more situation-independent language than meat foods (Davis & Papies, 2022; Papies, Johannes, et al., 2020). In other words, meat foods were presented more like diet-congruent “ingroup” foods in the current experiment. This pattern of language may discourage mainstream consumers from making sustainable food choices, by framing meat foods as the more rewarding choice. Indeed, taste expectations are considered to have a much larger influence on food choice (Boesveldt & de Graaf, 2017) than other factors, such as environmental concerns.

The current findings also show that mainstream consumers associate plant-based foods with more situation-independent features than meat foods, which are instead described with more consumption and reward features. Considering the positive relationship found between consumption and reward features and behavioral outcome measures, this suggests that appealing representations of meat foods can predict subsequent food choice. Future approaches to promote plant-based foods should avoid heavy use of situation-independent features which do not draw upon previous rewarding experiences that motivate consumption. In fact, research has found that taste-focused labeling, in comparison with health-focused labeling, can increase plant-based food selection by 29% (Turnwald et al., 2019), or even 38% (Turnwald & Crum, 2019). By presenting plant-based foods in terms of their rewarding features, eating simulations are more likely to occur—increasing desire and the probability of consumption. This could thus strengthen the associations of enjoyment with these foods, contributing to more sustainable eating habits.

### Theoretical Implications and Future Research

Our research provides support for a grounded cognition perspective on the relationship between food representations and consumption behavior (Papies & Barsalou, 2015). The frequency of typical consumption is likely to increase the number of consumption episodes to draw upon when cued, which may impact the production of rewarding food representations. Our findings are consistent with the idea that retrieving rewarding memories of past consumption experiences increases the attractiveness, consumption intentions and actual consumption of a food product, which is more likely to occur for frequently consumed, diet-congruent foods.

In addition, an ingroup-outgroup dimension emerged in our research that exists between people with different dietary patterns (Rosenfeld, 2018). One important determinant of readiness to reduce one’s meat intake is the social beliefs about people who do not consume meat (Branković & Budžak, 2021). Considering the salient vegan identity (Rosenfeld, 2019) attached to the consumption of plant-based foods, this may explain the reluctance to eat sustainable alternatives among mainstream consumers and thus the lack of rewarding representations for these diet-incongruent foods. For vegans, research has shown their attitudes toward omnivores are significantly more negative than vice versa (Pabian et al., 2022), which manifests in our findings through the number of negative, socio-political features used by vegans for meat foods. This suggests that the polarization between omnivores and vegans is conveyed even when presenting participants with dish names alone.

However, our research also provides evidence for dietary intergroup similarities. Previous literature suggests that health, animal welfare, and environmental concerns are common eating motivations among vegans (Hopwood et al., 2020). Despite this, Cliceri and colleagues (2018) found that vegans value the dimension of food pleasure as equally important as omnivores despite opposing dietary behaviors. Critically, our findings confirm that vegans are not “taste martyrs”; like omnivores, they use more rewarding features when asked to generate representations for diet-congruent foods that they frequently consume. Highlighting the similarities between these polarized groups, and their shared expectation of pleasure from eating, may help overcome polarization and remove barriers from mainstream consumers shifting toward sustainable diets.

Future research should consider examining the learning of food representations from the perspective of grounded cognition. Participants could be asked to list features before and after consuming a dish, to assess whether consumption and reward features are shaped by consumption. Furthermore, measuring actual consumption at different time points (e.g., 1 week, 1 month, and 3 months) might give richer insights into consumption effects over time. This may also help establish the causal mechanisms involved in the relationship between food representations and behavioral outcomes, which could not be addressed in the current study.

Future directions should also explore how these cognitive processes shift among consumers in the process of changing their dietary identities from omnivores to meat reducers, to flexitarians and beyond. Are those with more rewarding representations of plant-based foods more likely to become vegan, or does the process of dietary change toward veganism influence rewarding representations of plant-based foods? As described above, the patterns in the findings reported here probably capture both processes, such that participants may have become vegan because they found plant-based foods relatively appealing, and they then learned or updated their representations of plant-based foods to be increasingly more rewarding, helping them to sustain their dietary pattern. Nonetheless, greater exploration of these processes is needed. Furthermore, research could focus on how exposure to societal discourse about vegans shape food representations among mainstream consumers, in particular for plant-based foods. Other approaches investigating social influences on food representations, such as the influence of dietary intergroup contact, may reveal effective strategies to overcome negative perceptions of the outgroup and “their” foods. In addition, measuring the relationship between negative representations of meat foods and disgust sensitivity responses (Becker & Lawrence, 2021) may also be a key direction to explore among those with shifting dietary preferences.

### Strengths and Limitations

This is the first experiment of its kind to measure feature listing responses, desire and consumption behavior among different dietary groups. However, the self-reports of retrospective consumption behavior may have been inaccurate or biased (Hagger et al., 2015), compared with observational data. Future research should investigate the relationship between diet, food representations, and consumption behavior without relying on retrospective self-report. Furthermore, we included the same 20 foods for all participants, some of which some participants had not consumed before. Future research could improve on this by presenting idiosyncratically-selected stimuli, such that participants are familiar with all stimuli. In addition, the plant-based dish names were chosen to be representative of how plant-based foods are often labeled in real-world settings, that is, “plant-based,” “meat-free,” or “vegan” (Bacon et al., 2018). Consequently, some of our plant-based dish names were less specific than others (e.g., “vegan lasagne” vs. “lentil daal”), which may have prompted participants to list more situation-independent features, especially ingredient and content information. Nonetheless, we controlled for individual dishes in our models. Finally, our research only included data from U.K. residents, which may limit the generalizability to other populations. However, the strong societal polarization within the United Kingdom (Duffy et al., 2019) made this a suitable context for this project.

### Conclusion

In this paper, we found that participants use more consumption and reward features to describe diet-congruent foods than diet-incongruent foods, and these features predict desire, intentions, and future consumption. Representations of diet-congruent “ingroup” foods for both omnivores and vegans were characterized by short-term reward, while representations of diet-incongruent “outgroup” foods focused on abstract information and long-term consequences among omnivores, and social and political factors among vegans. Conceptualizing plant-based foods in terms of features separate to the consumption situation may impact mainstream consumer willingness to try sustainable alternatives. This work provides insights into the cognitive representations of foods that contribute to societal polarization around shifting diets to mitigate the climate emergency.
